# Effects of Regioisomerism on the Antiproliferative Activity of Hydroxystearic Acids on Human Cancer Cell Lines

**DOI:** 10.3390/molecules27082396

**Published:** 2022-04-07

**Authors:** Natalia Calonghi, Carla Boga, Patrizia Nitti, Dario Telese, Silvia Bordoni, Giovanna Farruggia, Fioretta Asaro, Martina Grandi, Chiara Zalambani, Gabriele Micheletti

**Affiliations:** 1 Department of Pharmacy and Biotechnology, University of Bologna, Via San Donato 15, 40127 Bologna, Italy; giovanna.farruggia@unibo.it (G.F.); martina.grandi11@unibo.it (M.G.); zalachiara@hotmail.it (C.Z.); 2 Department of Industrial Chemistry ‘Toso Montanari’, Alma Mater Studiorum Università di Bologna, Viale del Risorgimento 4, 40136 Bologna, Italy; dario.telese2@unibo.it (D.T.); silvia.bordoni@unibo.it (S.B.); gabriele.micheletti3@unibo.it (G.M.); 3 Department of Chemical and Pharmaceutical Sciences, University of Trieste, Via L. Giorgieri 1, 34127 Trieste, Italy; pnitti@units.it (P.N.); fasaro@units.it (F.A.); 4 Health Sciences and Technologies Interdepartmental Center for Industrial Research (CIRI SDV), University of Bologna, 40126 Bologna, Italy; 5 National Institute of Biostructures and Biosystems, Via delle Medaglie d’Oro 305, 00136 Rome, Italy

**Keywords:** hydroxystearic acids, cancer, quantum phase imaging, ptychography

## Abstract

A series of regioisomers of the hydroxystearic acid (**HSA**) was prepared, and the effect of the position of the hydroxyl group along the chain on a panel of human cancer cell lines was investigated. Among the various regioisomers, those carrying the hydroxyl at positions 5, 7, and 9 had growth inhibitor activity against various human tumor cell lines, including CaCo-2, HT29, HeLa, MCF7, PC3, and NLF cells. **10-HSA** and **11-HSA** showed a very weak effect. **8-HSA** did not show inhibitory activity in all cell lines. The biological role of **7-HSA** and **9-HSA** is widely recognized, while little is known about the effects of **5-HSA**. Therefore, the biological effects of **5-HSA** in HeLa, HT29, MCF7, and NLF cell lines were investigated using the Livecyte’s ptychography technology, which allows correlating changes in proliferation, motility, and morphology as a function of treatment at the same time. **5-HSA** not only reduces cell proliferation but also induces changes in cell displacement, directionality, and speed. It is important to characterize the biological effects of **5-HSA**, this molecule being an important component of fatty acyl esters of hydroxy fatty acids (FAHFA), a class of endogenous mammalian lipids with noticeable anti-diabetic and anti-inflammatory effects.

## 1. Introduction

Hydroxy fatty acids and their derivatives are compounds of growing interest in many scientific areas. In industry, they are widely used as lubricants [1,2,3], surfactants [4,5], plasticizers [6], additives in coatings and paintings [7,8], components of detergents, cosmetics [9,10], flavors [11], and foods [12,13,14]. They are ubiquitous in the biological world and are mainly found in triacylglycerols, membrane phospholipids, waxes, cerebrosides, and other lipids [15,16,17].

Among them, hydroxystearic acids have been widely studied for a long time, especially 12-hydroxystearic acid, due to its economical relevance and easy accessibility, it being produced from castor oil [18]. At present, the interest of biologists has been directed toward less investigated positional isomers, owing to their specific activity. Indeed, a recent study reports on the effect of some regioisomeric hydroxystearic acids as agonists of peroxisomal proliferator-activated receptors in increasing the anti-ageing potential of retinoids [19]. There has also been growing attention paid to fatty acyl esters of hydroxy fatty acids (**FAHFA**), a class of endogenous mammalian lipids with important effects on metabolism (e.g., anti-diabetic and anti-inflammatory effects) [20,21,22]. In these compounds, hydroxystearic acids (**HSA**s), mostly 5-hydroxystearic acid (**5-HSA**), 7-hydroxystearic acid **(7-HSA**), and 9-hydroxystearic acid **(9-HSA**), are esterified with palmitic acid (**PAHSA**) or other long-chain fatty acids. Moreover, the inhibitory activities of both enantiomers of **7-HSA** and **9-HSA** on A549, CaCo-2, and SF268 human cancer cell lines have been reported [23].

The biological activity of 9-hydroxystearic acid, an endogenous lipid able to inhibit cell growth in a series of cancer cell lines, has been, for a long time, the object of our research. **9-HSA** acts as a histone deacetylase HDAC1 inhibitor and, in human colorectal adenocarcinoma cells HT29, its effect is due to an arrest in the G0/G1 phase of the cell cycle [24,25,26,27,28,29,30]. Recently, our research focused on the development of delivering modes of **(*R*)-9-HSA** for possible biomedical applications, by loading it in biocompatible keratin nanoparticles [31], in magnetic nanoparticles [32], and in hydroxyapatite nanocrystals [33,34].

In the past, our group studied some positional homologs of hydroxystearic acid (namely **2-HSA**, **7-HSA**, **8-HSA**, **9-HSA**, **10-HSA**, and **12-HSA**) relevant to material chemistry [35,36,37], organic chemistry [38,39], and biology [40,41]. Considering the already known importance of both the carboxy- and the hydroxy- groups in inducing the antiproliferative activity of **9-HSA** [40], we planned to investigate the influence of the position of the hydroxy group along the stearic chain on the viability and motility of a panel of cancer cell lines. To this end, quantum phase imaging (QPI) based on ptychography was employed to evaluate cell number, confluence, cell dry mass, cell morphology, and motility [42,43].

**Figure 1:**
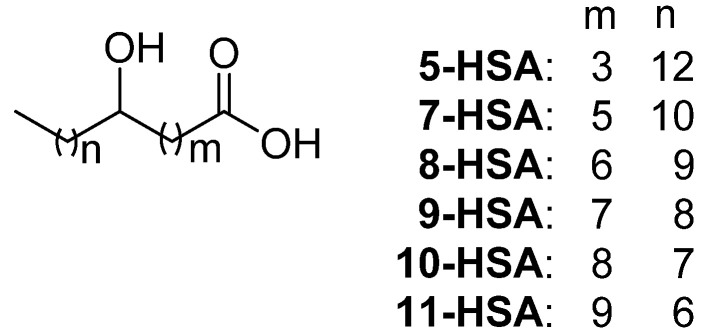
Hydroxystearic acids considered in current study.

Among all positional isomers of hydroxystearic acid, we decided to synthesize and investigate those shown in Figure 1, bearing the hydroxy group both on even and odd carbon atom. To the best of our knowledge, this is the first study on the anticancer activity of **5-HSA**, **8-HSA,** and **11-HSA**.

In this screening study, we opted for a simple synthetic chemical route able to obtain quickly the intended products with a good yield, starting from commercially available precursors. The outcomes of such synthesis are racemic mixtures, and due attention was exerted when comparing the antiproliferative activity with that reported in the literature for the pure enantiomeric species, owing to the relevance of HSAs’ chirality on their biological activity [23,44].

## 2. Results and Discussion

### 2.1. Synthesis of Regioisomeric Hydroxystearic Acids

**Scheme 1:**
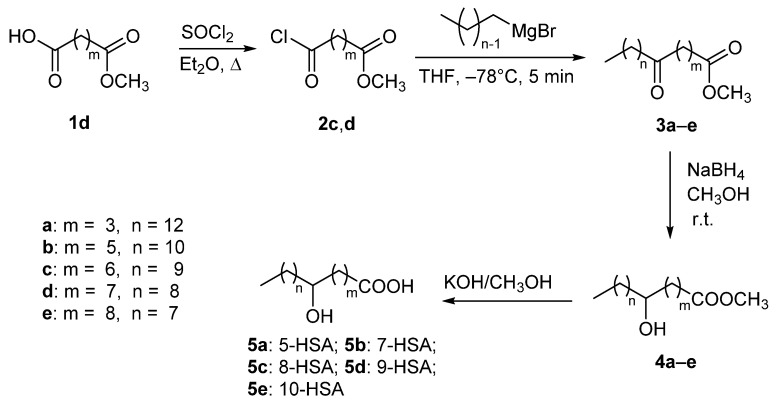
Synthetic routes to **HSA** derivatives **5a–e**.

The synthesis of the series of racemic **HSAs**, namely **5-HSA** (**5a** in Scheme 1), **7-HSA** (**5b** in Scheme 1), **8-HSA** (**5c** in Scheme 1), **9-HSA** (**5d** in Scheme 1), and **10-HSA** (**5e** in Scheme 1) was carried out starting from different points of the synthetic pathway depending on the commercial availability of the intermediates (Scheme 1). Keto derivatives **3c** and **3d**, precursors of **8-HSA** and **9-HSA**, respectively, were obtained by addition at low temperature of the appropriate Grignard reagent to the acyl chloride **2c** (commercially available) and **2d**, respectively. The latter was prepared from the azelaic acid monomethyl ester **1d** and thionyl chloride. Compounds **3c** and **3d**, together with commercially available **3a**, **3b**, and **3e,** were subjected to reduction in the keto group followed by hydrolysis of the esters functionality to give final compounds **5a–e**.

**Scheme 2:**
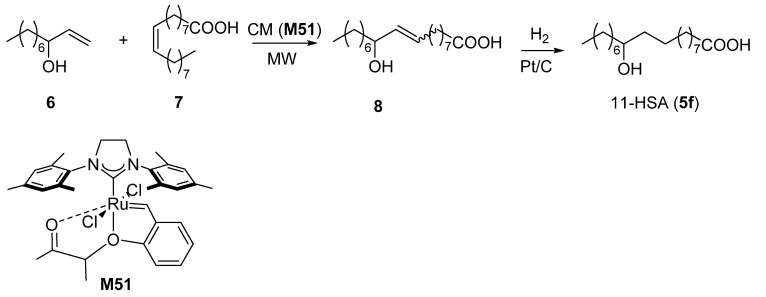
Synthesis of **11-HSA** (**5f**).

A different and novel procedure was developed for the synthesis of **11-HSA** (**5f**). As shown in Scheme 2, cross metathesis (CM) reaction [45] between 1-decen-3-ol (**6**) [46] and oleic acid (**7**) (1:3, respectively), carried out under microwave (MW) irradiation at 63 °C for 30 min with Grubbs Catalyst^®^ **M51** afforded a complex mixture of CM products [47] from which the unsaturated hydroxyacid (**8**) [48] in 20% yield was isolated by flash chromatography. After hydrogenation of compound **8** by an H-Cube system (ThalesNano Inc., cartridge containing 5% Pt/C) continuously producing H_2_ gas in small aliquots by electrolytic decomposition of H_2_O [49], the desired **11-HSA** (**5f**) was obtained in 47% yield.

### 2.2. Biological Activity

#### Cell Growth Inhibitory Effects of HSAs on Human Cancer Cell Lines

The growth inhibitory effects of six regioisomers of **HSA** on CaCo-2, HT29, HeLa, MCF7, PC3, and NLF cells were evaluated using the MTT assay, and the results are shown in Table 1.

Interestingly, the in vitro potency is not only influenced by the position of the hydroxy group in the chain but also by the characteristics of the individual cell lines.

In general, **5-HSA**, **7-HSA**, and **9-HSA** showed statistically significant inhibitory potency, while **10-HSA** and **11-HSA** exhibited a very weak effect. **8-HSA** showed no inhibitory activity in all cell lines.

**7-HSA** was found to exhibit the highest growth inhibitory potency (IC_50_ values 14.7, 26.6, 21.4, 24.3, and 24.9 μM for HT29, HeLa, MCF7, PC3, and NLF cells, respectively), **5-HSA** (IC_50_ values 25.1 and 22.1 μM for CaCo-2 and HeLa cells, respectively), **11-HSA** (IC_50_ values 27.6, 35.8, and 29.7 μM for CaCo-2, MCF7, and NLF cells, respectively), all being clearly more potent than their regioisomer, **9-HSA**.

Prior to this study, we had reported that **9-HSA** upregulates p21WAF1 in HT29 cancer cells [24] and inhibits cell growth in human colon cancer cells by targeting histone deacetylase 1 [25].

Other authors demonstrated that both (*R*) enantiomers of **7-HSA** and **9-HSA** induce the arrest of the cell cycle but do not promote apoptosis [23]. Furthermore, no growth inhibitory effect of 7-PAHSA, 9-PAHSA, and oleic acid-hydroxy stearic acid (9-OAHSA) was observed, excluding the possibility that such an effect was caused by FAHFAs generated in cells after HSA treatment. These observations indicate that acylation of the hydroxyl group in a fat chain destroys growth inhibitory potency [23].

While the biological role of **7-HSA** and **9-HSA** is widely recognized, little is known about the effects of **5-HSA**. For this reason, we decided to further investigate the biological effects of **5-HSA** in HeLa, HT29, MCF7, and NLF cell lines. To this end, Livecyte’s ptychography technology allows correlating changes in proliferation, motility, and morphology as a function of treatment at the same time.

**Figure 2:**
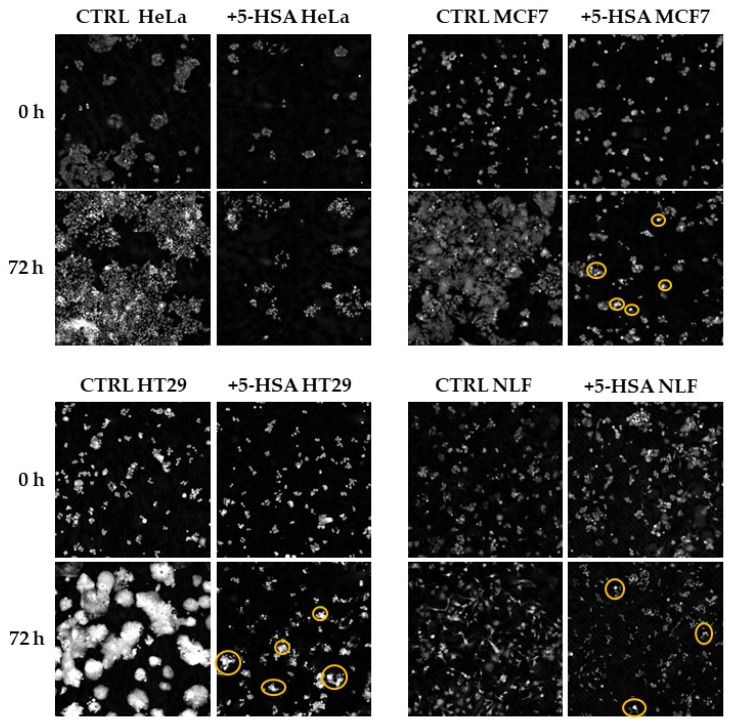
Quantitative phase images. Representative images at 0 and 72 h of HeLa, HT29, MCF7, and NLF control cells and treated with 50 μM **5-HSA**. Yellow circles indicate dense cells.

In Figure 2, the images of the cells at 0 and 72 h of treatment are reported. It is evident that **5-HSA** inhibits cell proliferation, but the cells after 72 h of treatment are still adherent. However, several cells with a rounded morphology are present in all the samples. In MCF7 and HT29, dense cells (indicated in yellow circles), which appear whiter than the others, seem to indicate the induction of apoptosis.

**Figure 3:**
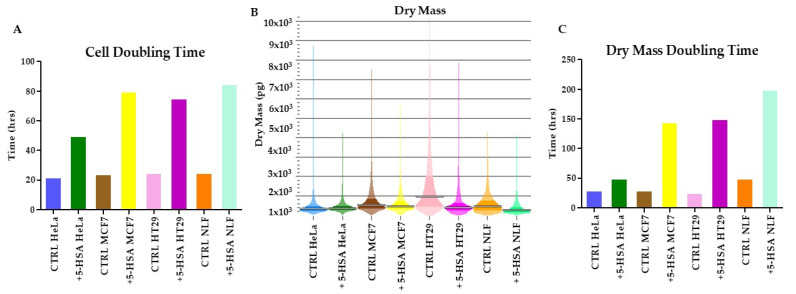
Effect of 50 μM **5-HSA** treatment on cell proliferation and morphology. (**A**) Histogram plot illustrating median cell doubling time. (**B**) Distribution of the cellular dry mass for HeLa, MCF7, HT29, and NLF cells, treated and control. (**C**) Histogram plot illustrating dry mass doubling time of cells, treated and control.

In Figure 3A–C, the doubling times calculated as cell counts and as dry mass of a typical experiment are reported.

In Figure 3A, the graph clearly shows how **5-HSA** more strongly reduces cell proliferation rate and increases cell doubling time in MCF7 (from 24 to 80 h), HT29 (from 25 to 75 h), and NLF cells (from 25 to 75 h), than in HeLa (from 22 h to 50 h).

The graph 3B shows how **5-HSA** decreases the total dry mass accumulation per field of view in all cell lines, except in HeLa.

The dry cell mass is an important parameter; in fact, it represents the total mass of all cellular components, including proteins, lipids, carbohydrates, and DNA, excluding water.

Graph 3C shows how the differences between **5-HSA** groups and control in dry mass doubling time are higher for MCF7 (from 30 to 145 h), HT29 (from 25 to 150 h), and NLF (from 50 to 200 h) than for Hela (from 30 to 50 h) cells. This indicates that, especially in MCF7, HT29, and NLF, treatment with **5-HSA** decreases proliferation and also that the cells are smaller but still alive and attached.

The minor growth inhibition caused by **5-HSA** on HeLa cells can be attributed to a specific cell behavior.

However, the differences of the doubling time of dry mass between **5-HSA** groups and control are more marked than those observed for the cell doubling time (Figure 3A,C). This indicates that, in these cells, the reduction in proliferation and biomass synthesis is most affected.

Instead, in HeLa, treatment with **5-HSA** shows a similar trend in median dry cell mass compared to control group. This indicates that in HeLa, individual cell growth (increase in biomass) increases, but cells do not proliferate. This could be interpreted as a consequence of cell cycle arrest in G1; however, further studies would be required to confirm such an interpretation.

Deregulation of cell motility can result in diseases, such as cancer, autoimmune disorders, neurological diseases, and chronic inflammation. Direct, non-invasive label-free imaging measurements of cell motility allow detecting cell speed, which is independent of factors such as proliferation, path directionality, and tortuousness [42].

**Figure 4:**
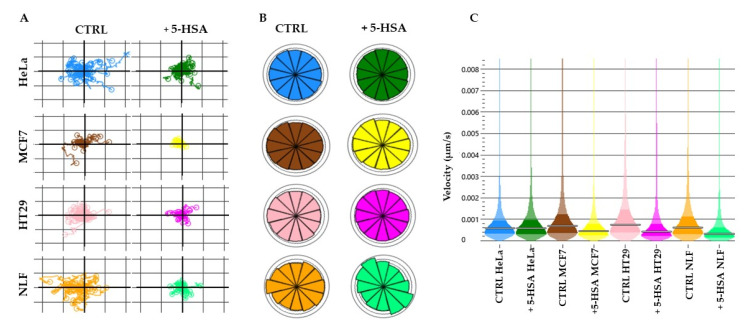
Analysis of cellular motility. (**A**) Average confinement ratio of HeLa, MCF7, HT29, and NLF cells over 72 h after treatment. (**B**) Directionality and (**C**) Average instantaneous velocity of HeLa, MCF7, HT29, and NLF cells over 72 h after treatment. Error bars represent the inter-quartile range.

In Figure 4, the plots for cell displacement (A), directionality (B), and velocity (C) are reported. Treatment reduces the cellular movements in MCF7 cells, while the directionality is more affected in NLF cells, which move less and in a more asymmetric way. The instantaneous velocity is diminished in all cells, except in HeLa. Motility measurements indicate that HeLa are less affected by **5-HSA**, showing speed and directionality similar to control; only displacement is slightly reduced. On the other hand, MCF7 are blocked in their movements, both in displacement and speed. It is interesting that only NLF show a modified directionality with respect to the other cell lines. Motility measurements confirm the heterogeneity of the response of these cells to **5-HSA**, even if this reduced parameter could suggest a minor metastatic capability.

## 3. Materials and Methods

### 3.1. Chemical Synthesis

#### 3.1.1. General

The nuclear magnetic resonance spectra (^1^H NMR and ^13^C NMR,) were recorded at 25 °C on the Varian Spectrometer Mercury 400 and 400-MR (Varian, Palo Alto, Santa Clara, CA, USA) both operating at 400 MHz for proton. Frequencies are reported in Hz, and the chemical shifts were referenced to the solvent (CDCl_3_, δ = 7.27 and 77.0 ppm for ^1^H and ^13^C NMR, respectively). Signal multiplicities were established by DEPT-135 experiments. ESI-MS spectra were recorded using a Waters 2Q 4000 instrument (Waters Corporation, Milford, MA, USA). Melting points were measured on a Büchi apparatus (Stone, Staffs, UK) and are not corrected. For flash chromatography (FC), silica gel 0.037–0.063 mm (Merck KGaA, Darmstadt, Germany) was used as stationary phase. Thin layer chromatography (TLC) was carried out on silica gel 60 (Fluka Analytical, Buchs, Switzerland), and the spots were revealed using an aqueous solution of (NH_4_)_6_MoO_24_ (2.5%) and (NH_4_)_4_Ce(SO_4_)_4_ (4%) in 10% H_2_SO_4_. Microwave irradiations were performed by an Anton Paar Monowave 400 (Anton Paar GmbH Graz, Graz, Austria) instrument. Continuous hydrogenation was performed with an H-cube Mini-Plus Thales-Nano system (Thalesnano Inc, Budapest, Hungary) using a cartridge containing 5% Pt/C, 15 bar inlet pressure, 1 mL/min flow rate, 25 °C temperature, 30 bar H_2_ pressure, 2 runs of a 0.01 M solution of compound 2 in ethanol. Nonanedioic acid 1-methyl ester (**1d**), methyl 8-chloro-8-oxooctanoate (**2c**), methyl 5-oxooctadecanoate (**3a**), methyl 7-oxooctadecanoate (**3b**), methyl 10-oxooctadecanoate (**3e**), n-decylmagnesium bromide (1.0 M in diethyl ether), thionyl chloride, and Grubbs Catalyst^®^ M51 were purchased from Sigma-Aldrich (Milan, Italy). 1-Decen-3-ol (**6**) was obtained by vinylation of n-octanal with vinyl magnesium bromide according to the literature [46].

7-Hydroxyoctadecanoic acid (**5b**), 8-hydroxyoctadecanoic acid (**5c**), 9-hydroxyoctadecanoic acid (**5d**), and 10-hydroxyoctadecanoic acid (**5e**) were prepared according to the procedure previously reported by us [38,41] and shown in Scheme 1, and their chemico-physical data agree with those reported there.

#### 3.1.2. Synthesis of Methyl 5-Hydroxyoctadecanoate (**4a**)

Sodium borohydride (0.152 g, 4.0 mmol) was added portion-wise to a solution of methyl 5-oxooctadecanoate (**3a**, 0.625 g, 2.0 mmol)) in methanol (20 mL), and the mixture was stirred at room temperature. The reaction progress was monitored by TLC (light petroleum/diethyl ether: 7/3). Once the absence of the starting keto derivative was verified, the reaction mixture was treated with water (5 mL) and extracted with ethyl acetate (3 × 10 mL). After extraction with brine, the collected organic layers were dried over anhydrous MgSO_4_ and filtered. The solvent was removed ‘in vacuo’ and after purification by flash chromatography of the residue, pure **4a** (0.520 g, 1.66 mmol, 83%) was obtained. Characterization data of compound **4a** agree with those reported in the literature [50,51,52]. Some ^13^C NMR signals were assigned on the basis of ref. [52].

#### 3.1.3. Synthesis of 5-Hydroxyoctadecanoic Acid (**5-HSA**, **5a**)

Methyl 5-hydroxyoctadecanoate (**4a**, 0.450 g, 1.43 mmol) was dissolved (with a slight warming) in 25 mL of a solution of KOH in methanol (10% *w*/*v*) and stirred for 2 h at room temperature. The reaction was monitored by TLC (eluent: petroleum ether/diethyl ether 7/3). The solvent was completely removed under vacuum, and the light-yellow solid was dissolved in water and acidified with 6 M HCl until precipitation of the acid as a white solid. This mixture was extracted with ethyl acetate (3 × 20 mL), and the organic layers were dried over anhydrous MgSO_4_. After filtration, the solvent was removed under vacuum. The white residue was recrystallized from methanol to give pure **5a** (0.300 g, 1 mmol, 70% yield). White solid, m.p.: 80.6–81.8 °C (Lit. [53]: 81 °C), ^1^H NMR (CDCl_3_, 25 °C, 400 MHz) δ (ppm): 3.65–3.57 (m, 1H), 2.40 (t, *J* = 7.4 Hz, 2H), 1.88–1.76 (m, 1H), 1.76–1.64 (m, 1H), 1.59–1.36 (m, 4H), 1.36–1.20 (m, 22H), 0.88 (t, *J* = 6.9 Hz, 3H); ^13^C NMR (CDCl_3_, 25 °C, 100.5 MHz) δ (ppm): 178.0 (C-1), 71.6 (C-5), 37.5 (C-6), 36.5 (C-4), 33.6 (C-2), 31.9 (C-16), 29.68 (C-14), 29.66 (C-13), 29.65 (C-12), 29.64 (2 signals overlapped, C-10, C-11), 29.60 (C-9), 29.59 (C-8), 29.3 (C-15), 25.6 (C-7), 22.7 (C-17), 20.8 (C-3), 14.1 (C-18); ESI-MS (m/z): 301 [M + H^+^], 323 [M + Na^+^], 339 [M + K^+^].

#### 3.1.4. Synthesis of 11-Hydroxyoctadecanoic Acid (**11-HSA**, **5f**)

##### Synthesis of 11-Hydroxy-9-octadecenoic Acid (**8**)

In a G10 vial for Anton Paar Monowave 400 instrument, 1-decen-3-ol (**6**, 0.165 g, 1.06 mmol), oleic acid (**7**, 0.895 g, 3.17 mmol), and Grubbs Catalyst^®^ M51 (6.93 mg, 0.01 mmol) dissolved in 1 mL of CH_2_Cl_2_ were added. The temperature was set to 63 °C, and the mixture was irradiated for 30 min under stirring. The brown crude reaction mixture was immediately purified by flash chromatography (ethyl acetate/light petroleum, gradient from 5% up to 14% and adding 0.5 mL of glacial acetic acid every 100 mL of eluent), compound **8** (0.063 g, 0.21 mmol) was isolated in 20% yield. All spectroscopic data are in accordance with the literature [48], Rf = 0.42 light petroleum/ethyl acetate 80:20 + 1 mL of glacial acetic acid every 100 mL. Characterization data of compound **8** agree with those reported in the literature [48].

##### Synthesis of 11-Hydroxyoctadecanoic Acid (**11-HSA**, **5f**)

Compound **8** (0.045 g, 0.15 mmol) was dissolved in 15 mL of ethanol, and the solution was hydrogenated with an H-cube Mini-Plus Thales-Nano system using a cartridge containing 5% Pt/C, 15 bar inlet pressure, 1 mL/min flow rate, 25 °C temperature, 30 bar H_2_ pressure, 2 runs. After evaporation of the solvent, the crude reaction mixture was dissolved in ethyl acetate and extracted with a phosphate-buffered solution at pH = 7.4 in order to remove octadecanedioic acid formed as a by-product. The organic phase was dried on anhydrous Na_2_SO_4_ and evaporated to afford **11-HSA**.

11-Hydroxystearic acid (**5f**, **11-HSA**): 47% yield (0.020 g, 0.07 mmol). M.p.: 68.3–68.6 °C (Lit. [54]: 71–72 °C), ^1^H NMR (400 MHz, CDCl_3_) δ (ppm) 3.59 (bs, 1H, H-11), 2.7–2.2 (bs, 2H, OH), 2.35 (t, *J* = 7.5 Hz, 2H), 1.69–1.57 (m, 2H), 1.36 (m, 26H), 0.88 (t, *J* = 6.9 Hz, 3H); ^13^C NMR (100.5 MHz, CDCl_3_) δ 179.32 (C-1), 72.10 (C-11), 37.42 (C-12), 37.38 (C-10), 33.94 (C-2), 31.82 (C-16), 29.65 (C-14), 29.59 (C-8), 29.46 (C-7), 29.28 (2 signals overlapped, C-6, C-15), 29.14 (C-5), 28.99 (C-4), 25.63 (C-13), 25.57 (C-9), 24.64 (C-3), 22.64 (C-17), 14.08 (C-18). ESI-MS (m/e): 301 [M + H]^+^, 323 [M + Na]^+^, [M + K]^+^.

### 3.2. Biology

#### 3.2.1. Cell Culture and Treatments

The human colorectal adenocarcinoma (CaCo-2), human colorectal adenocarcinoma (HT29), human cervical cancer (HeLa), human breast cancer (MCF7), human caucasian prostate adenocarcinoma (PC3), and human neuroblastoma (NLF) cell lines were purchased from American Type Culture Collection (ATCC, Manassas, VA, USA). Cells were cultured in RPMI 1640 medium (Labtek Eurobio, Milan, Italy), supplemented with 10% FCS (Euroclone, Milano, Italy) and 2 mM L-glutamine (Sigma-Aldrich, Milano, Italy), at 37 °C, and a 5% CO_2_ atmosphere. The compounds were dissolved in ethanol in a 30–40 mM stock solution. In cell treatments, the final ethanol concentration never exceeded 0.2%.

#### 3.2.2. MTT Assay

Cells were seeded at 1.5 × 10^4^ cells/well in a 96-well culture plastic plate (Sarsted, Milan, Italy), and after 24 h growth they were exposed to increasing concentrations of **5-HSA** (**5a**), or **7-HSA** (**5b**), or **8-HSA** (**5c**), or **10-HSA** (**5e**), or **11-HSA** (**5f**) (from 0.010 μM to 500 μM) solubilized in RPMI 1640 medium. MTT assay was performed according to Ref [55]. In brief, after 24 h treatment, the culture medium was replaced with 0.1 mL of 3-(4,5-dimethylthiazolyl-2)-2,5-diphenyltetrazolium bromide (MTT, Sigma-Aldrich) dissolved in PBS at the concentration of 0.2 mg/mL, and samples were incubated for 2 h at 37 °C. The absorbance at 570 nm was measured using a multi-well plate reader (Tecan, Männedorf, Switzerland), and data were analyzed by Prism GraphPad software and expressed as IC_50_ μM.

#### 3.2.3. Quantitative Phase Image (QPI) Microscopy

Ptychography uses multiple diffraction patterns collected from spatially overlapping regions of the samples to form QPI images. QPI is a label-free technique based on different methods, which allows obtaining the phase information of light passing through the cell. QPI techniques quantify the extent of phase delay introduced by the sample and record it as pixel values within the generated image. Its intensity is due to the thickness and the refraction index of the cell, which depends on the biomolecule composition and organization within the cell [56,57,58,59].

Quantitative phase image (QPI) microscopy assay was performed using the Livecyte microscope (Phase Focus Limited, Sheffield, UK) according to the manufacturer’s indications. In brief, HT29, HeLa, MCF7, and NLF were seeded in a 96-well plate (Sarsted, Milan, Italy) at 4 × 10^3^ per well. After 24 h, cells were treated with **5-HSA** 50 μM in six replicates, and the images were acquired every 60 min for 3 days using a 10× objective lens (0.25 NA), at 37 °C and 5% CO_2_. Data were analyzed using the Cell Analysis Toolbox software (Phase Focus Limited, Sheffield, UK) to evaluate cell growth, doubling times, and motility.

### 3.3. Statistical Analysis

All experiments were performed in triplicate and repeated at least three times. Results were averaged, and the standard deviation was calculated. To determine statistical significance, unpaired two-tailed Student’s *t*-test was used between 2 different independent groups. A *p*-value below 0.05 was considered significant.

## 4. Conclusions

The regioisomeric series of hydroxystearic acids, namely **5-HSA**, **7-HSA**, **8-HSA**, **9-HSA**, **10-HSA**, and **11-HSA** were synthesized in good yield through simple multistep synthetic procedures, and their growth inhibitory effects were evaluated on a panel of cancer cell lines.

In general, isomers with the hydroxy group bound to odd carbon atoms (**5-HSA**, **7-HSA**, and **9-HSA**) showed significant inhibitory potency, while **10-HSA** and **11-HSA** exhibited a very weak effect, and **8-HSA** showed no inhibitory activity in all cell lines. Since little is known about its effects, we focused particular attention on **5-HSA**, demonstrating that it shows cell growth inhibitory properties. Quantum phase imaging allowed us to investigate **5-HSA** effects more deeply on the four cell lines tested, since treatment influences important cellular parameters, such as dry biomass, morphology, and motility.

The possibility of evaluating cell growth both as the number of cells and as biomass allows us to show that **5-HSA** not only reduces cell proliferation but also affects cell morphology. In fact, the cells are smaller and more rounded, suggesting the induction of apoptosis.

The study of cell migration and cell motility is of great importance to understand disease. Cell migration can be the cause of cancer progression and metastases formation. Cell motility is essential in many aspects of biology, e.g., immune regulation, tissue regeneration, and embryogenesis. In our studies, we demonstrated that **5-HSA** affects cell motility, in particular displacement, directionality, and velocity.

Furthermore, the observation that half maximal inhibitory concentration of **5-HSA** in HeLa (IC_50_ 22.1 μM) is lower than in MCF7 (IC_50_ 46.4 μM), HT29 (IC_50_ 51.3 μM) and NLF (IC_50_ 38.5 μM) indicates how the potency of the molecule is affected by the characteristics of the individual cell lines.

An important final consideration is that this is a preliminary work, performed using the racemic mixture of the considered HSA derivatives. As demonstrated by Kokotou M. et al. [23], it is important to consider the different biological activity of the enantiomers, and this will be the object of future studies.

## Figures and Tables

**Table 1:** IC_50_ (μM) values of the HSA derivatives in the analyzed tumor cell lines.

	5-HSA IC_50_	7-HSA IC_50_	8-HSA IC_50_	9-HSA IC_50_	10-HSA IC_50_	11HSA IC_50_
CaCo2 R square	25.1 μM 0.8889	42.5 μM 0.9731	> 100μM	32.6 μM 0.9472	68.3 μM 0.9730	27.6 μM 0.9944
HT29 R square	51.3 μM 0.9575	14.7 μM 0.9255	> 100μM	30.7 μM 0.9635	77.2 μM 0.9048	56.9 μM 0.8918
HeLa R square	22.1 μM 0.9886	26.6 μM 0.9703	> 100μM	26.9 μM 0.9321	41.7 μM 0.8073	31.5 μM 0.8981
MCF7 R square	46.4 μM 0.9820	21.4 μM 0.9447	> 100μM	49.1 μM 0.9866	38.7 μM 0.9599	35.8 μM 0.9681
PC3 R square	31.6 μM 0.9516	24.3 μM 0.9183	> 100μM	23.4 μM 0.9499	34.0 μM 0.8826	61.4 μM 0.9610
NLF R square	38.5 μM 0.9971	24.9 μM 0.8366	> 100μM	33.1 μM 0.9960	74.6 μM 0.9887	29.7 μM 0.9841

## Data Availability

Not applicable.

## Sample Availability

Samples of some compounds are available from the authors.
